# Letter to the editor regarding ‘Performance of novel biomarkers for prediction of diabetic kidney disease in patients with diabetes mellitus’

**DOI:** 10.1080/07853890.2026.2612781

**Published:** 2026-01-21

**Authors:** Kaiyang Xu, Yutong Qian, Chen Zhao

**Affiliations:** Shanghai University of Traditional Chinese Medicine, Shanghai, Pudong New Area, China

Dear Editor

We recently read with great interest the study by Zou et al. titled ‘Performance of novel biomarkers for prediction of diabetic kidney disease in patients with diabetes mellitus’ published in Annals of Medicine [1]. This study provides valuable insights into diabetic kidney disease (DKD) risk stratification by evaluating seven promising biomarkers, filling a critical gap in non-invasive early detection of this devastating complication. However, we would like to share some thoughts and suggestions for further discussion to enhance the depth and generalizability of the findings.

The two-phase design, combining cross-sectional and prospective cohorts, strengthens the validity of the findings. We particularly appreciate the identification of urinary MCP-1 as a standalone top performer in both DKD diagnosis (AUC = 0.722) and prediction, and the four-biomarker panel (GDF15, MCP-1, ANGPTL4, FGF23) achieving an impressive AUC of 0.873 for 2-year DKD risk. This suggests potential advantages over traditional markers such as UACR and eGFR [2]. However, we note a few points worthy of discussion. First, the single-center setting and relatively small prospective sample (*n* = 141) may limit generalizability. External validation across diverse populations would further confirm the model’s robustness. Second, the low prescription rate of guideline-recommended therapies (SGLT-2 inhibitors, RAS inhibitors) in the cohort raises questions about potential confounding, as these agents impact both DKD progression and biomarker levels [3,4].

Despite these limitations, this study makes a significant contribution to DKD research. The machine learning analysis, comparing seven algorithms, adds practical value by tailoring model selection to clinical priorities—LightGBM for high recall and SVM for precision. This work paves the way for personalized risk assessment and early intervention, which is crucial given the rising global burden of DM and DKD. We commend the authors for their rigorous approach and meaningful findings. Further research with larger multi-center cohorts and longer follow-up is warranted to validate these biomarkers and translate them into routine clinical practice.

## Data Availability

Data sharing is not applicable to this article as no data were created or analyzed in this study.
